# Choline Deficiency Causes Colonic Type II Natural Killer T (NKT) Cell Loss and Alleviates Murine Colitis under Type I NKT Cell Deficiency

**DOI:** 10.1371/journal.pone.0169681

**Published:** 2017-01-17

**Authors:** Shintaro Sagami, Yoshitaka Ueno, Shinji Tanaka, Akira Fujita, Hiroaki Niitsu, Ryohei Hayashi, Hideyuki Hyogo, Takao Hinoi, Yasuhiko Kitadai, Kazuaki Chayama

**Affiliations:** 1 Department of Medicine and Molecular Science, Hiroshima University, Hiroshima, Japan; 2 Department of Endoscopy, Hiroshima University Hospital, Hiroshima, Japan; 3 Department of Gastroenterological and Transplant Surgery, Applied Life Sciences, Institute of Biomedical & Health Sciences, Hiroshima University, Hiroshima, Japan; 4 Department of Gastroenterology, Hiroshima General Hospital, Hiroshima, Japan; 5 Department of Surgery, Institute for Clinical Research, National Hospital Organization Kure Medical Center and Chu-goku Cancer Center, Hiroshima, Japan; 6 Department of Life Sciences, Prefectural University of Hiroshima, Hiroshima, Japan; Kurume University School of Medicine, JAPAN

## Abstract

Serum levels of choline and its derivatives are lower in patients with inflammatory bowel disease (IBD) than in healthy individuals. However, the effect of choline deficiency on the severity of colitis has not been investigated. In the present study, we investigated the role of choline deficiency in dextran sulfate sodium (DSS)-induced colitis in mice. Methionine-choline-deficient (MCD) diet lowered the levels of type II natural killer T (NKT) cells in the colonic lamina propria, peritoneal cavity, and mesenteric lymph nodes, and increased the levels of type II NKT cells in the livers of wild-type B6 mice compared with that in mice fed a control (CTR) diet. The gene expression pattern of the chemokine receptor CXCR6, which promotes NKT cell accumulation, varied between colon and liver in a manner dependent on the changes in the type II NKT cell levels. To examine the role of type II NKT cells in colitis under choline-deficient conditions, we assessed the severity of DSS-induced colitis in type I NKT cell-deficient (Jα18^-/-^) or type I and type II NKT cell-deficient (CD1d^-/-^) mice fed the MCD or CTR diets. The MCD diet led to amelioration of inflammation, decreases in interferon (IFN)-γ and interleukin (IL)-4 secretion, and a decrease in the number of IFN-γ and IL-4-producing NKT cells in Jα18^-/-^ mice but not in CD1d^-/-^ mice. Finally, adaptive transfer of lymphocytes with type II NKT cells exacerbated DSS-induced colitis in Jα18^-/-^ mice with MCD diet. These results suggest that choline deficiency causes proinflammatory type II NKT cell loss and alleviates DSS-induced colitis. Thus, inflammation in DSS-induced colitis under choline deficiency is caused by type II NKT cell-dependent mechanisms, including decreased type II NKT cell and proinflammatory cytokine levels.

## Introduction

Inflammatory bowel diseases (IBDs) such as Crohn’s disease (CD) and ulcerative colitis (UC) result from uncontrolled intestinal immune responses toward commensal microflora and dietary antigens [1], as well as uncontrolled genetic abnormalities [2]. We previously reported that the course of CD was less severe among patients with CD and NAFLD, with a longer surgery-free interval and higher rate of remission. The better prognosis in CD patients with NAFLD was evident even in patients with low BMI [3]. Previous studies have implicated choline deficiency in the development of nonalcoholic fatty liver disease (NAFLD) [4, 5]. CD and UC patients have lower levels of choline and its derivatives than healthy individuals. Choline and glycerophosphorylcholine are significantly lower in the mucosa of UC and CD patients experiencing active phases than in healthy controls and UC and CD patients in remission [6]. Choline is an essential nutrient required for the formation of phosphatidylcholine (PC) [7]. Decreased levels of choline, PC, and glycerophosphorylcholine are caused by an increased use of choline owing to inflammation in CD and UC patients [8]. Changes in trimethylamine (TMA) caused by gut microflora induce perturbations in choline metabolism, which contributes to nonalcoholic steatohepatitis (NASH) by reducing choline bioavailability [9, 10]. Very low-density lipoproteins (VLDLs) are also downregulated in UC remission patients compared with active patients [7], as PC biosynthesis is required for the formation of VLDLs in the liver [11]. Deficiencies in choline and PC also predispose patients to NAFLD *via* VLDL deficiency [12].

The main food groups contributing to choline intake are meat, milk, grain, eggs and their derived products, composite dishes, and fish [13]. These foods may be restricted in IBD patients [14, 15]; therefore, diet may also affect choline intake. Short bowel syndrome and total parenteral nutrition affect choline metabolism [4, 16]. Gut microbial metabolism of choline results in the production of TMA. The TMA-producing status of the gut microbiota should be considered when making recommendations about choline intake requirements [17–19].

Although many risk factors contribute to choline deficiency in IBD patients, it is unknown whether choline deficiency affects the severity of colitis. Therefore, we investigated the role of a methionine-choline-deficient (MCD) diet in dextran sulfate sodium (DSS)-induced colitis in mice. An MCD diet has been previously shown to lead to fat accumulation in the liver [20, 21]. Moreover, hepatic NK1.1^+^ CD3^+^ T cells (type I and type II natural killer T [NKT] cells) have been found to be elevated in mice fed an MCD diet [22, 23]. It is believed that type I NKT cells play a protective role in DSS-induced colitis, whereas colonic type II NKT cells play a pathogenic role [24]. The results of the current study suggest that choline deficiency leads to the loss of IFN-γ-producing type II NKT cells, alleviating DSS-induced colitis.

## Materials and Methods

### Mice

Specific pathogen-free C57BL⁄6 (B6) mice were purchased from CLEA Japan (Tokyo, Japan). B6-Jα18^-/-^ and B6-CD1d^-/-^ mice were originally generated by Dr. M. Taniguchi (Chiba University, Chiba, Japan) and Dr. Luc Van Kaer (Vanderbilt University, Nashville, TN), respectively. All mice were housed under specific pathogen-free conditions in microisolator cages in the animal facility at Hiroshima University, and only male mice (9–14 weeks of age) were used. Mice were divided into two groups: those fed an MCD diet and those fed a CTR diet. This study was performed in strict accordance with the recommendations in the Guide for the Care and Use of Laboratory Animals of the Hiroshima University Animal Research Committee and the AVMA Guidelines on Euthanasia. The protocol described below was approved by the Committee on the Ethics of Animal Experiments of Hiroshima University (Permit Number: UK28-179). All mice were housed in a specific pathogen-free facility in 12 h light-dark cycles with access to water and food *ad libitum*, and the health of the mice was monitored every day. Cervical dislocation euthanasia of all adult mice and pups was performed after pentobarbital sedation (40 mg/kg).

### NAFLD model

One group of mice was fed an MCD diet (F2-MCD, Oriental Yeast, Tokyo, Japan) for the indicated length of time. The MCD diet was supplied with corn oil (10%, w/w), and no fish oil was added. The other group was fed a CTR diet (MF, Oriental Yeast). Following fixation of the mouse livers with 10% formalin/phosphate-buffered saline, the livers were sectioned and stained with hematoxylin and eosin (H & E) for histological examination. Liver injury was quantified by measuring serum enzyme activities of alanine aminotransferase (ALT) using a Hitachi (Tokyo, Japan) 7180 Automatic Analyzer with L-Type ALT.J2 (Wako, Osaka, Japan) and H & E staining using a previously published scoring system [25]. Serum triglycerides (TG) were measured with L-Type Triglyceride M (Wako).

### DSS-induced colitis model

Mice were treated with 2% DSS (MW 5 kDa; Wako Chemical Co., Osaka, Japan) in drinking water for 7 days (4–6 mice per group). Body weight was measured every day, beginning on the first day of administration of DSS (day 0). Differences in body weight from day 0 were represented as a percentage of the body weight.

A rigid-bore endoscope (AE-R16150, AVS Co., Ltd., Japan) was used to assess spontaneous bleeding from perianal lesions and transparency of the colonic wall as the endoscope entered the rectum. Spontaneous bleeding was defined as naturally occurring mucosal hemorrhaging not associated with traumatic endoscopy, and transparency was defined as the ability to visualize intramural blood vessels in the colon and those of other surrounding viscera [26].

The disease activity index (DAI; combined score of weight loss, stool consistency, and bleeding) was used to assess the severity of colitis, based on a previously published scoring system [27]. Scoring of the mice was performed 7 days after the first administration of DSS. Colonic tissues were removed and opened longitudinally. The length of the colon was measured after exclusion of the cecum and prior to division for histology. The tissues then were rolled concentrically and embedded in paraffin. Sections were stained with H & E and coded for blind microscopic assessment of inflammation (i.e., DSS-induced colitis). Colitis was scored on three parameters according to morphological criteria, as described previously [28]. Severity of inflammation was scored as follows: 0, rare inflammatory cells in the lamina propria; 1, increased number of granulocytes in the lamina propria; 2, confluence of inflammatory cells extending into the submucosa; and 3, transmural extension of the inflammatory infiltrate. Crypt damage was scored as follows: 0, intact crypts; 1, loss of the basal one-third; 2, loss of the basal two-thirds; 3, entire crypt loss; 4, change of epithelial surface with erosion; and 5, confluent erosion. Ulceration was scored as follows: 0, absence of ulcers; 1, one or two foci of ulceration; 2, three or four foci of ulceration; and 3, confluent or extensive ulceration. Values were added to give a maximum potential histological score of 11.

### Preparation of mononuclear cells

Tissue lymphocytes from the colonic lamina propria, liver, and spleen were prepared as described previously [29, 30]. Mesenteric lymph node cells were passed through a 70-μm filter and washed with Ca^++^Mg^++^-free Hanks balanced salt solution. Peritoneal cells were collected by washing the peritoneal cavity with 10 mL of ice-cold phosphate-buffered saline.

### Cell culture for cytokine production in the colonic lamina propria

Cells were harvested, transferred to 96-well plates (2 × 10^5^ cells per well), and stimulated *in vitro* with 100 ng/mL lipopolysaccharide (LPS; Sigma, St Louis, MO, USA) for 24 h at 37°C and 5% CO_2_. Supernatants were collected and stored at -80°C until further analysis. Concentrations of cytokines, including interferon (IFN)-γ, interleukin (IL)-10, and IL-4, in culture supernatants were measured with ELISA MAX sets (BioLegend, San Diego, CA, USA), according to the manufacturer’s instructions. All samples were analyzed in triplicate.

### *In vivo* migration

In the present study, lamina propria cells (2×10^6^ cells) from B6-Jα18^-/-^ mice were labeled with PKH26GL Red Fluorescent Cell Linker Dye (Sigma-Aldrich, Tokyo, Japan) and were injected intraperitoneally into healthy B6-Jα18^-/-^ mice (day 0) to analyze *in vivo* migration. Specific organs were evaluated and compared on day 7 after transfer between the MCD and CTR mice. To this end, solid organs were cut into sections, and the lumen of the colon was opened. The samples were then analyzed by *in situ* fluorescence microscopy using a Zeiss LSM 510 laser scanning microscopy system (Carl Zeiss Inc., Thornwood, NY, USA), as described previously [31]. PKH-labeled lamina propria cells were analyzed by flow cytometry after cell-surface staining with antibodies against NK1.1 (Miltenyi Biotec, Bergisch Gladbach, Germany) and CD3 (BD Pharmingen, San Diego, CA, USA).

### Flow cytometry

The following fluorophore-conjugated antibodies were used for cell-surface staining: CD3 (BD Pharmingen, San Diego, CA, USA), CD3e (BD Pharmingen), NK1.1 (BD Pharmingen), B220 (BD Pharmingen), CXCR6 (BioLegend), CD11b (BD Pharmingen), CD11c (BD Pharmingen), Gr-1 (BioLegend), and F4/80 (BioLegend). All antibodies were used at empirically determined dilutions in PBS. CD1d tetramer (MBL International, Woburn, MA, USA) was incubated with α-galactosylceramide (α-GalCer) for 16 h at 37°C, according to the manufacturer’s instructions, prior to staining. Antibodies used for intracellular staining included IFN-γ (BioLegend) and IL-4 (BD Pharmingen). For flow cytometric analysis of cytokine production, lymphocytes were first stimulated *in vitro* with 1 μg/mL LPS or 50 μg/mL phorbol myristate acetate + 1000 μg/mL ionomycin in the presence of monensin (BD Biosciences, San Jose, CA, USA) at 37°C for 5 h. Cells were then stained with antibodies against the indicated cell-surface markers, followed by staining of cytokines with an intracellular staining kit (BD Biosciences) in the presence of CD16/32 Fc-receptor blocker (BD Pharmingen). Debris and dead cells were excluded based on forward scatter, side scatter, and positive staining with DAPI (Dojindo Molecular Technologies, Rockville, MD, USA) or the ZombieNIR Fixable Viability Kit (BioLegend). Flow cytometric analysis was performed with FlowJo (Treestar, Ashland, OR, USA).

### Real-time PCR

Tissue lymphocytes were isolated from the colonic lamina propria and liver, as described above. RNA was isolated using an RNeasy Mini kit (QIAGEN, Tokyo, Japan) according to the manufacturer’s instructions. cDNA was synthesized from 500 ng of total RNA using a Reverse Transcription Kit (QIAGEN), and quantitative PCR was performed with SYBR Green MasterMix (QIAGEN) using LightCycler (Roche, Basel, Switzerland) according to the manufacturer’s recommended protocol. Samples were normalized to β-actin expression in lymphocytes isolated from the colon of wild-type mice. The primer sequences are listed in S1 Table.

### Statistical analysis

Data are expressed as the mean ± standard error (SE). Statistical significance was analyzed using Prism ver. 6.0 (GraphPad Software, San Diego, CA, USA). Unpaired *t*-tests were performed to compare two groups. To compare multiple groups, one-way analysis of variance (ANOVA) using Tukey’s post hoc test was performed. *P* < 0.05 was considered statistically significant. Body weight data were analyzed by one-way ANOVA with repeated measures over time.

## Results

### Hepatic lipid accumulation is induced by MCD diet

**Fig 1:**
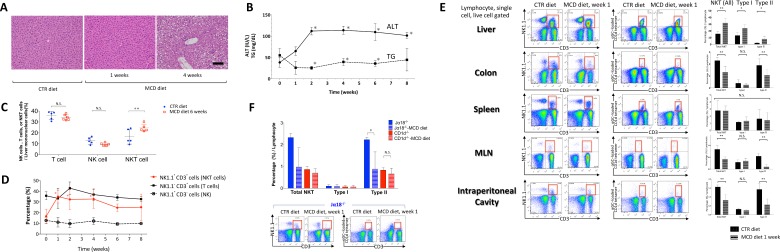
Methionine-choline deficient (MCD) diet induces nonalcoholic steatohepatitis (NASH) and natural killer T (NKT) cell accumulation. (A) Representative images of hematoxylin and eosin (H & E) staining of mice fed the MCD diet for 1 week and 4 weeks or the control (CTR) diet. Scale bar, 100 μm. (B) Serum alanine aminotransferase (ALT) and triglyceride (TG) levels in MCD diet-fed mice over 8 weeks. *N* = 4–6, *P < 0.05 vs. MCD diet week 0, paired *t*-test. (C) Intrahepatic NK1.1^+^ T (NKT) cells were evaluated by flow cytometry in MCD diet-fed mice over 6 weeks. *N* = 5–6, **P < 0.01, Student’s *t*-test. (D) Intrahepatic NKT cells, natural killer (NK) cells, and NK1.1^-^ T (T) cells were measured by flow cytometry in MCD diet-fed mice for 1, 2, 4, 6, or 8 weeks. *N* = 2–6, *P < 0.05, vs. MCD 0 week, paired *t*-test. (E) Effects of 1 week of MCD diet on NKT cell subsets in mononuclear cells of the liver, colonic lamina propria, spleen, mesenteric lymph node (MLN), and intraperitoneal cavity in wild-type mice. Black and hatched bars represent NKT type I and type II cells, respectively. *N* = 4, *P < 0.05, **P < 0.01, Student’s *t*-test. (F) Effects of 1 week of MCD diet on type I and type II NKT cell subsets in the lamina propria in Jα18^-/-^ and CD1d^-/-^ mice, as measured by flow cytometry. *N* = 4, *P < 0.05, Student’s *t*-test. All data represent the mean ± standard error of the mean (s.e.m.).

As expected, mice fed the MCD diet for 4 weeks exhibited steatohepatitis and higher macroscopic lipid content than CTR-fed mice (Fig 1A). The levels of alanine aminotransferase increased and the levels of triglycerides decreased in the MCD mice from week 2 to week 8 (Fig 1B). We also studied the immune cell subsets in mice with NAFLD. Consistent with previous reports [22, 23], the number of hepatic NK1.1+CD3+T cells (type I and type II NKT cells) increased over weeks 1–6 under the MCD diet (Fig 1C and 1D). The ratio of NK1.1^-^ CD3^+^ T cells (T cells) to NK1.1^+^ CD3^-^ cells (NK cells) remained unchanged until week 8 of the MCD diet (Fig 1D). The numbers of hepatic B220^+^ cells and γδ T cells were also unchanged (data not shown). These findings suggest that choline deficiency affected hepatic NKT cells. The proportion of hepatic macrophages, dendritic cells (DCs), and Gr-1^+^ cells increased, consistent with previous reports [32, 33]. Significantly fewer NKT cells were found in the colonic lamina propria, spleen, mesenteric lymph nodes, and peritoneal cavity than in the liver of MCD mice, which was also consistent with previous observations [34]. Under conditions of choline deficiency, the number of both type I and type II NKT cells increased in the liver of MCD mice, and the numbers and ratios of type II NKT cells were significantly lower in the colonic lamina propria, mesenteric lymph nodes, and peritoneal cavity of the MCD mice compared with those in the CTR mice. However, whereas the numbers of NKT cells without the α-GalCer-loaded Cd1d tetramer (type II NKT cells) were reduced in the colonic lamina propria after one week of the MCD diet, those of NKT cells with the α-GalCer-loaded Cd1d tetramer (type I NKT cells) remained unchanged (Fig 1E), and this tendency was also observed after DSS-induced colitis in Jα18^-/-^ and CD1d^-/-^ mice (S1 Fig). Therefore, we analyzed the frequency of NKT cells in the colonic lamina propria after MCD feeding under conditions of type I NKT cell deficiency (Jα18^-/-^ mice) and type I and type II NKT cell deficiency (CD1d^-/-^ mice). We observed that only colonic type II NKT cell levels were significantly reduced (Fig 1F) in mice on the MCD diet. These data indicate that choline deficiency decreases the accumulation of type II NKT cells in the colonic lamina propria.

### MCD diet up-regulate CXCR6 and EP4 expression and alter distribution of colonic type II NKT cells

**Fig 2:**
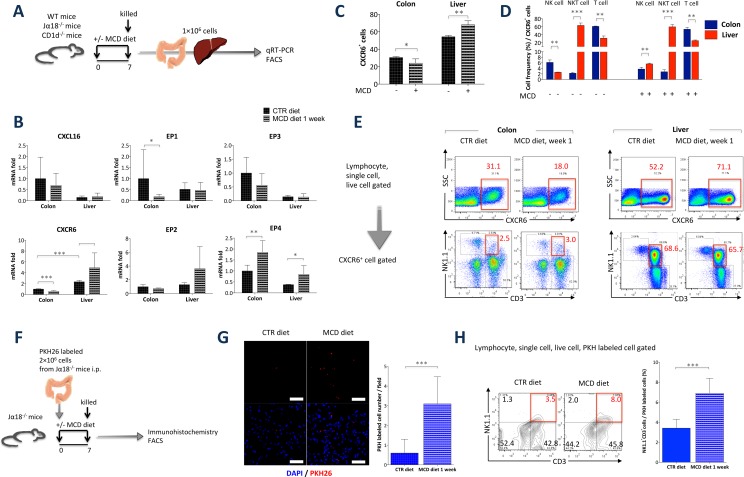
Level of CXCR6 was elevated in the liver, and the redistribution induced by the MCD diet was altered under choline-deficient conditions. (A) Experimental setup. (B) Gene expression levels of *CXCR6*, *CXCL16*, *EP1*, *EP2*, *EP3*, and *EP4* (normalized to β-actin), as assessed by quantitative PCR, in hepatic mononuclear cells and colonic lamina propria cells isolated from mice fed the MCD diet for 1 week compared with that in mice fed the CTR diet. (C-E) Representative flow cytometry plots and frequency of CXCR6^+^ population in liver and colonic lamina propria in WT mice. *N* = 4, *P < 0.05, **P < 0.01, ***P < 0.001 Student’s *t*-test. (F) Experimental setup. (G) Immunofluorescence DAPI staining (×40) of liver from Jα18^-/-^ mice with or without MCD diet at day 7 after PKH-labeled cells injection. PKH26 (red) and DAPI (blue) were expressed, respectively. PKH-labeled cells per field were enumerated. Data from 10 representative fields from four individual mice are plotted as mean ± s.e.m. ***P < 0.001, Student’s *t*-test. (scale bar = 50 μm) (H) Expression of NK1.1 and CD3 on PKH-labeled single live cells harvested from the liver at day 7 after injection of PKH-labeled cells. *N* = 4, ***P < 0.001, Student’s *t*-test.

Our experiments indicated that choline deficiency changes the localization of NKT cells in the liver and colon. CXCR6 and CXCL16 play critical roles in NKT cell activation, cytokine generation, and NKT cell localization [35]. Prostaglandin E2 (PGE2) is also associated with NKT cell anergy [36]. To test whether chemokine or PGE2 change is associated with the localization of NKT cells, we assessed the gene expression levels of *CXCR6*, *CXCL16*, and *PGE2* receptors (*EP1*, *EP2*, *EP3*, and *EP4*) in hepatic and colonic lamina propria mononuclear cells by quantitative PCR. Hepatic CXCR6 and EP4 were upregulated in mice fed the MCD diet compared with that in mice fed the CTR diet. On the contrary, colonic CXCR6 was downregulated in mice fed the MCD diet compared with that in mice fed the CTR diet. Colonic CXCR6 expression was lower than hepatic expression (Fig 2B). These findings are consistent with the results obtained for NKT cell localization in the liver and colon in mice fed the MCD diet. Significant difference in the intrahepatic and colonic CXCR6^+^ population of cells was observed between WT and CD1d^-/-^ mice (Fig 2C–2E, S2 Fig). Choline deficiency in CD1d^-/-^ mice also increased and decreased with hepatic and colonic CXCR6^+^ cell frequency retrospectively (S2 Fig), suggesting that choline deficiency affects the distribution of CXCR6^+^ NK cells, besides that of NKT cells. These data suggest that the chemokine receptor CXCR6 may influence NKT cell localization in choline deficiency.

To investigate whether the MCD diet induced a redistribution of colonic type II NKT cells, we adoptively transferred PKH-labeled Jα18^-/-^ lamina propria cells into Jα18^-/-^ mice at day 0 intraperitoneally and assessed the redistribution of PKH-labeled type II NKT cells at day 7 (Fig 2F). The transfer of PKH-labeled Jα18^-/-^ lamina propria cells into Jα18^-/-^ mice resulted in a significant increase in Jα18^-/-^ mice fed the MCD diet compared with Jα18^-/-^ mice fed the CTR diet in liver (Fig 2G). Notably, transferring Jα18^-/-^ type II NKT cells into MCD Jα18^-/-^ mice resulted in a significant increase in the frequency of PKH-labeled lymphocytes, clearly demonstrating a causative impact of choline deficiency on hepatic type II NKT cell phenotype (Fig 2H). Furthermore, frequency of PKH-labeling in mesenteric lymph node and colonic lamina propria was significantly reduced, consistent with the results shown in Fig 1E (S3A and S3B Fig). Together these data indicate that hepatic type II NKT cell numbers were increased and colonic type II NKT cell numbers were decreased under choline deficiency.

### MCD diet attenuates DSS induced-colitis in mice with type II NKT cells

**Fig 3:**
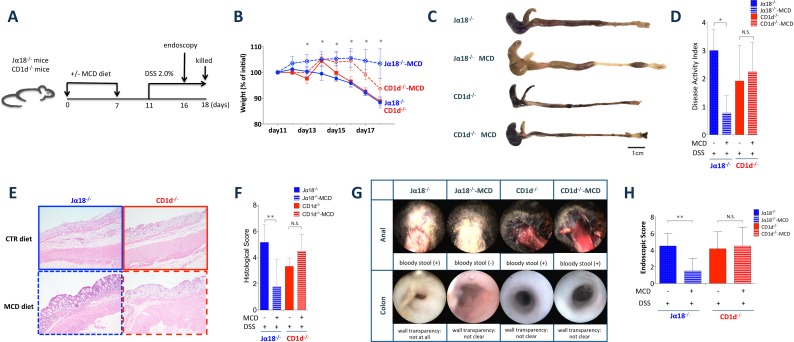
The MCD diet attenuates dextran sodium sulfate (DSS)-induced colitis under type I NKT cell deficiency. (A) Experimental setup. (B) Body weight (percentage of body weight relative to initial body weight) of Jα18^-/-^ and CD1d^-/-^ mice fed the MCD and CTR diets, measured after 2.0% DSS treatment. *N* = 4–6, *P < 0.05 (Jα18^-/-^ CTR vs. Jα18^-/-^ MCD). (C) Representative macroscopic photographs and (D) disease activity index (DAI) scores of colons of DSS-treated Jα18^-/-^ and CD1d^-/-^ mice fed the MCD or CTR diets. *N* = 4, *P < 0.05, Student’s *t*-test. (E) Histological examination (H & E staining) and (F) histological scores of the colons of Jα18^-/-^ and CD1d^-/-^ mice fed the MCD or CTR diets 7 days after 2.0% DSS treatment. Original magnification × 100. All data are presented as means ± SE. *N* = 6, *P < 0.05, Student’s *t*-test. (G) Endoscopic evaluation and (H) endoscopic evaluation score of colorectal health in Jα18^-/-^ and CD1d^-/-^ mice. *N* = 6, *P < 0.05, **P < 0.01.

Since the frequency of colonic type II NKT cells was significantly reduced in mice on the MCD diet, we assessed the role of type II NKT cells in DSS-induced colitis under conditions of choline deficiency. We compared the development of colitis in MCD and CTR Jα18^-/-^ mice to test whether the loss of type II NKT cells regulated DSS-induced colitis. Moreover, we also compared the development of colitis in MCD or CTR CD1d^-/-^ mice to determine whether the effect remains without type II NKT cells (Fig 3A). Body weight loss was reduced in Jα18^-/-^-MCD mice but not in CD1d^-/-^ MCD mice (Fig 3B). Shorter colon lengths and bloody stool were also attenuated in Jα18^-/-^ MCD mice. Histopathology revealed milder historical crypt damage and ulceration and less inflammatory cell infiltration in the colons of Jα18^-/-^-MCD mice (Fig 3E). There were significant differences in this regard between Ja18^-/-^mice fed the CTR diet and those fed the MCD diet (Fig 3F). Furthermore, endoscopic analysis revealed that Jα18^-/-^ MCD mice exhibited milder colitis on day 5 of DSS induction. In contrast, the endoscopic scores of CD1d^-/-^ MCD mice remained unchanged (Fig 3G). These data suggest that MCD mitigates DSS-induced colitis through an associated reduction in type II NKT cells.

### Colonic type II NKT cell loss contributes to the attenuation of DSS-induced colitis

**Fig 4:**
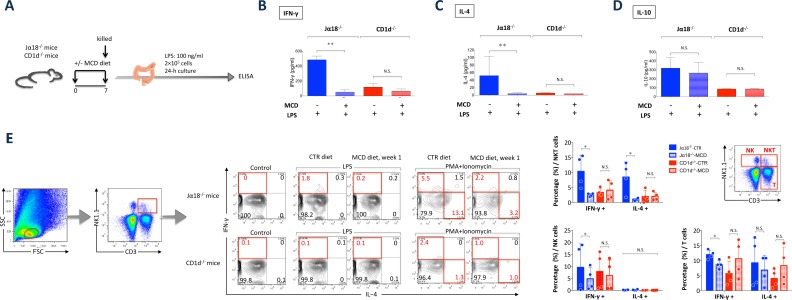
MCD diet reduces proinflammatory cytokine levels and the number of type II NKT cells in type I NKT cell-deficient mice. (A) Experimental setup. (B) IFN-γ, (C) IL-4, and (D) IL-10 production by colonic lamina propria mononuclear cells following treatment with 100 ng/mL lipopolysaccharide (LPS) at 24 h, as analyzed by ELISA. *N* = 4, *P < 0.05, Student’s *t*-test. (E) Three subsets of colonic lamina propria cells separated using NK1.1 and CD3. *Ex vivo* intracellular IL-4 and IFN-γ production stimulated by LPS (1 μg/mL) or phorbol myristate acetate (PMA) + ionomycin for 5 h in NKT cells in the lamina propria in MCD vs. CTR diet-fed mice.

We evaluated the role of NKT cells in DSS-induced colitis in colonic lamina propria cells. As reported previously, colonic type II NKT cells play a pathogenic role in DSS-induced colitis [24]. Consistent with this mechanism, levels of the proinflammatory cytokines IFN-γ and IL-4 were elevated in Jα18^-/-^ mice relative to the levels in CD1d^-/-^ mice fed the CTR diet (Fig 4B and 4C). However, both IFN-γ and IL-4 levels were reduced after the mice were fed the MCD diet, whereas IL-10 production was unaffected by the MCD diet (Fig 4B, 4C and 4D). Further phenotypic profiling showed that colonic NKT cells in Jα18^-/-^ CTR mice constitutively synthesized IFN-γ and IL-4 in the lamina propria compared with Jα18^-/-^ MCD mice (Fig 4E). These data collectively demonstrate that lamina propria type II NKT cells secrete IFN-γ and IL-4, which exacerbates DSS-induced colitis. However, the frequency of type II NKT cells and their IFN-γ and IL-4 production is reduced by the MCD diet (Figs 1F, 4E and 4F). The frequencies of IFN-γ-producing T cells and NK cells were also reduced by the MCD diet (Fig 4F). Interestingly, the MCD diet caused a decrease in proinflammatory cytokine levels as well as a reduction in the number of colonic type II NKT cells, suggesting an inhibitory effect of choline deficiency on this cell population.

### Colonic type II NKT cell adoptively transfer contributes to the attenuation of DSS-induced colitis

**Fig 5:**
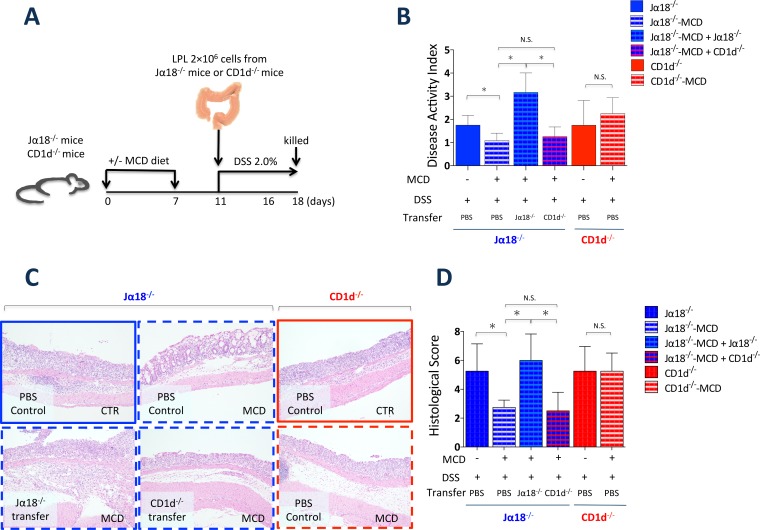
Adaptive transfer of Jα18-CTR lamina propria cells, but not CD1d-CTR lamina propria cells, restored colonic inflammation in DSS-induced colitis. (A) Experimental setup. (B) Disease activity index (DAI) scores of colons of DSS-treated Jα18^-/-^ and CD1d^-/-^ mice fed the MCD or CTR diets, and transferred with lamina propria cells from Jα18^-/-^ and CD1d^-/-^ mice. *N* = 4, *P < 0.05, Student’s *t*-test. (C) Histological examination (H & E staining) and (D) histological scores of the colons of Jα18^-/-^ and CD1d^-/-^ mice fed the MCD or CTR diets 7 days after 2.0% DSS treatment. Original magnification × 100. All data are presented as means ± SE. *N* = 4, *P < 0.05, Student’s *t*-test.

To investigate the role of type II NKT cells in DSS-induced colitis with choline deficiency, we adoptively transferred Jα18^-/-^-CTR lamina propria cells in Jα18^-/-^-MCD mice (Fig 5A). The adoptive transfer of Jα18^-/-^-CTR lamina propria cells exacerbated colitis in Jα18^-/-^-MCD mice. These data suggest that type II NKT cell loss protects against DSS-induced colitis and that type II NKT cells have a pathogenic role in colitis, consistent with the results of previous studies. [24, 37]

## Discussion

We examined the role of choline in DSS-induced colitis in mice to determine whether the immunopathogenesis of colitis is affected by choline deficiency. The results presented here highlight a new role for choline, demonstrating that choline deficiency causes colonic type II NKT cell loss and alleviates DSS-induced colitis (S3A and S3B Fig). Although choline deficiency has been implicated in a mouse model of NASH [10, 20, 38, 39], to the best of our knowledge, this is the first study in which choline deficiency has been shown to be associated with the suppression of colitis.

Consistent with previous results [23], we have shown that feeding of mice with the MCD diet gives rise to NASH over 4 weeks, characterized by lipid accumulation, a progressive increase in hepatic triglyceride content, and transaminase release. In a recent study, choline deficiency de-regulated lipid metabolism by generating higher levels of mitochondrially derived reactive oxygen species in the liver [40]. Choline deficiency was also found to cause marked hepatosteatosis and increased hepatic NKT cell numbers and function in both humans and rodents [38, 41–43]. Hepatic NKT cells accumulated following MCD feeding over 1–4 weeks, whereas NK cells and T cells were unaffected in wild-type mice over 8 weeks of MCD feeding. Since MCD time course and liver fibrosis depend on the proportion of hepatic NKT cells, the finding that a choline-deficient diet causes hepatic NKT cell loss is controversial [22, 38, 44]. In our study, the frequency of hepatic NKT cells peaked over weeks 1–4 after MCD feeding in wild-type mice. Most previous studies analyzed the NKT cells population after 4 weeks, when NAFLD was initiated. Thus, our finding that 1 week of MCD diet challenge promotes NKT cell accumulation in the liver is novel. Moreover, our results suggest that these NKT cells accumulate prior to the onset of histological hepatosteatosis. Based on these findings, we speculate that choline deficiency affects the NKT cell population earlier than observed in previous studies [22, 23, 38, 44], suggesting that NKT cells induce NASH. Several reports have indicated that both type I and type II NKT cells induce NASH in other NASH models (e.g., *ob/ob* mice, mice fed a high fat diet) [45–48]. Therefore, NKT cell play an important role in the long-term development of NASH. Moreover, the MCD diet causes an increase in the NKT cell population at a very early stage of NASH.

Surprisingly, the frequency of NKT cells, especially type II NKT cells, was reduced in the colonic lamina propria, spleen, mesenteric lymph node, and intraperitoneal cavity after 1 week of MCD feeding in wild-type mice. The MCD diet caused an increase in the frequency of NKT cells in the liver; in contrast, the MCD diet caused a decrease in the frequency of these cells in the colon in wild-type mice. It is possible that the distribution of NKT cells varies among different organs with the MCD diet. In addition, the proportion of type II NKT cells was significantly reduced in the colonic lamina propria of Jα18^-/-^-MCD mice, similar to wild-type mice, but not in CD1d^-/-^-MCD mice. These results suggest that the mechanism regulating type II NKT cells loss in choline deficiency is independent of type I NKT cells. Type II NKT cells play a pathogenic role in DSS-induced colitis and UC [24, 37, 49, 50]. These alterations in type II NKT frequency indicate that choline deficiency affects the development of colitis.

The chemokine receptor CXCR6 and its ligand CXCL16 play critical roles in homeostasis and activation of type I and II NKT cells. In a previous study, IFN-γ production by hepatic NKT cells was impaired and the cell numbers of NK1.1^+^ NKT cells decreased in CXCR6^-/-^ mouse [35]. Furthermore, the ability of CXCR6^-/-^ NKT cells to accumulate in the liver of the recipient mouse was reduced [51]. In this study, choline deficiency increased the intrahepatic mRNA level of CXCR6, and decreased that in the colon. Since CXCR6 has been reported to be expressed on NKT cells [52], it was not clarified whether change in the CXCR6 level was the cause or consequence of NKT cell migration. However, protein level of CXCR6 was also affected in colon and liver under type I and II NKT cell deficiency as well as in WT mice. And, the source of CXCR6 molecules was hepatic NKT cells. These results suggest that choline deficiency affects CXCR6 expression regardless of the existence of NKT cells. However, we cannot totally exclude the possibility that change in the CXCR6 level was the consequence of NKT cell migration. Prostaglandin E2 (PGE2) in intestinal mucus-derived proinflammatory nanoparticles induced an anergy-like state in hepatic NKT cells, which produce IFN-γ and IL-4 in a PGE2 E-type prostanoid 2 (EP2)/E-type prostanoid 4 receptor (EP4)-mediated manner [36]. EP4 is one of the four receptors for PGE2, and it plays important roles in choline metabolism and prevention of colitis [53–55]. In particular, EP4 regulates phosphatidylcholine production. [53] Thus, an overall EP-4 upregulation may be caused by choline deficiency. The association between EP4 and NAFLD has not been clarified. However, PGE2, via activation of EP4 receptors, functions as an endogenous anti-inflammatory mediator in mouse adipose tissue, and targeting EP4 may mitigate adipose tissue inflammation in a high-fat diet model by suppressing a greater variety of chemokines [56].

In this study, we demonstrated that choline deficiency attenuates DSS-induced colitis under conditions of type I NKT cell deficiency. Since type II NKT cells have a pathogenic role, three possibilities can explain these observations: First, lysophosphatidylcholine exhibits pro-inflammatory effects [57]. Isoforms C18:0 and C16:0 of LPC, which display a choline head group, are the most potent at activating sulfatide-reactive type II NKT cells [57]. In our study, LPC stimulated mononuclear cells in the colonic lamina propria to produce IFN-γ (data not shown), consistent with a previous report. Because serum LPC levels are reduced in MCD mice [58], type II NKT cells may not be able to become activated in these mice. Second, both intestinal epithelial cells and DCs functionally express CD1d on their cell surfaces [59], and LPC is sensed by DCs to modulate their function [60]. Type II NKT cells exposed *in vivo* to low levels of CD1d expression may fail to contribute to the development of colitis in mice. Finally, lipid antigens from commensal or pathogenic bacteria, as well as self-lipids, may activate type II NKT cells, contributing to the pathogenesis of IBD [24, 48, 61]. For example, treatment of mice with broad-spectrum antibiotics attenuated colitis caused by type II NKT cells [24]. Choline deficiency may lead to the loss of CD1d-expressing cells, which deactivate type II NKT cells. Analysis of the gut microbiota following feeding with the MCD diet revealed that lactic acid bacteria levels were reduced in feces [62]. In the present study, we did not analyze choline metabolism, CD1d expression, or the microbiota of the mice; thus, the factors responsible for the regulation of colonic type II NKT cells require further study.

In this study, we demonstrated that IFN-γ and IL-4 production from lamina propria mononuclear cells was reduced in Jα18^-/-^-MCD mice but not in CD1d^-/-^-MCD mice, whereas the IL-10 levels remained unchanged. In addition, the amount of IFN-γ and IL-4 produced by NKT cells in the lamina propria was reduced in Jα18^-/-^-MCD mice but not in CD1d^-/-^-MCD mice. Thus, our results indicate that choline deficiency induces the loss and deactivation of colonic type II NKT cells in mice. We also found that the MCD diet improved colitis in Jα18^-/-^ mice but not in CD1d^-/-^ mice. Moreover, adoptive transfer of lamina propria cells from Jα18^-/-^ mice, but not from CD1d^-/-^ mice, aggravated colitis in Jα18^-/-^ mice with choline deficiency. Thus, our data suggest that choline deficiency reduces the number of type II NKT cells in the colon, leading to the dysregulation of type II NKT cells and attenuation of the severity of colitis.

However, alternative explanations for the effects of choline deficiency need to be considered. To address the possible impact of choline deficiency, studies using mouse models that are more resistant to NAFLD (e.g. BALB/c mice instead of B6 mice) [10] need to be performed. Further, using other type of colitis model (e.g. 2,4,6-trinitrobenzene sulfonic acid (TNBS)-induced colitis) will confirm reliability of our findings. Next, the relationship between choline deficiency and human NKT cells is not unknown. Hence, we should investigate how diet standardization and choline deficiency influence the development of IBD under conditions of choline deficiency.

In contrast, a protective role has been reported for type I NKT cells in the DSS-induced colitis model. In a previous study, activation of type I NKT cells by α-GalCer led to a significant improvement in DSS-induced colitis based on assessment of body weight, bleeding, diarrhea, and survival [63]. We have previously shown that repeated stimulation of type I NKT cells with α-GalCer altered the Th1/Th2 balance by reducing the Th1 response and improving the disease score in DSS-induced colitis [64], and administration of OCH, an α-GalCer analog, attenuated colonic inflammation by polarizing type I NKT cells to a Th2-type cytokine production profile [29]. IL-9-producing type I NKT cells protect against DSS-induced colitis through IFN-γ and IL-17A suppression, as well as IL-10 and TGF-β upregulation, depending on IL-4 production by type I NKT cells [65]. Consistent with these findings, our experiments revealed that Jα18^-/-^ mice suffered from aggravated DSS-induced colitis compared with wild-type mice (data not shown). Wild-type mice were not significantly affected by choline deficiency; thus, colonic type I NKT cells may not be affected by choline levels.

Type I NKT cells are deficient, both systemically and mucosally, in CD patients [66]. This is also true in Jα18^-/-^ mice. Reducing type II NKT cell stimulation by altering the lipids recognized may offer a way to modulate the activity of IBD and other diseases. The frequency of type II NKT cells is significantly increased in patients with active phase UC and other immune diseases [50], suggesting that this feature may be useful as a clinical biomarker of IBD and other diseases. The ability to regulate NKT cell levels with choline deficiency may aid in controlling the disease activity of IBD.

## Supporting Information

S1 Table Primers used for real-time PCR. (PDF)

S1 Fig (A) Effects of 1 week of MCD diet on type I and type II NKT cell subsets in the lamina propria of wild-type, Jα18^-/-^, and CD1d^-/-^ mice before DSS administration. *N* = 4, *P < 0.05, Student’s *t*-test. (B) Effects of 1 week of MCD diet on type I and type II NKT cell subsets in the lamina propria of wild-type, Jα18^-/-^, and CD1d^-/-^ mice after DSS administration. *N* = 4, *P < 0.05, Student’s *t*-test.
(PDF)

S2 Fig (A-C)Representative flow cytometry plots and frequency of CXCR6^+^ population in liver and colonic lamina propria in CD1d^-/-^ mice. *N* = 4, *P < 0.05, **P < 0.01, Student’s *t*-test.
(PDF)

S3 Fig (A-B) Immunofluorescence DAPI staining (×40) of the colon, spleen, mesenteric lymph node from Jα18^-/-^ mice with or without MCD diet at day 7 after injection of PKH-labeled cells. PKH26 (red) and DAPI (blue) were expressed. PKH-labeled cells per field were enumerated. Data from 10 representative fields from four individual mice are plotted as mean ± s.e.m. *P < 0.05, **P < 0.01, Student’s t-test. Scale bar = 50 μm.
(PDF)

S4 Fig Graphical abstract. (PDF)
