# Supplementary material for: Choline Deficiency Causes Colonic Type II Natural Killer T (NKT) Cell Loss and Alleviates Murine Colitis under Type I NKT Cell Deficiency
Source: PLoS One. 2017 Jan 17;12(1):e0169681. doi: 10.1371/journal.pone.0169681 (PMC5241147; doi:10.1371/journal.pone.0169681)
Supplement: S1 Table — (PDF) [file pone.0169681.s001.pdf]

S1 Table

Gene	bp	Forward primer	Reverse primer
Cxcl16	147	TGAACTAGTGGACTGCTTTGAGC	GCAAATGTTTTTGGTGGTGA
Cxcr6	490	TACGATGGGCACTACGAGGGAG	GCAAAGAAACCAACAGGGAGACCAC
Ep1	666	TTAACCTGAGCCTAGCGGAT	CGCTGAGCGTATTGCACACTA
Ep2	536	GTGGCCCTGGCTCCCGAAAGTC	GGCAAGGAGCATATGGCGAAGGTG
Ep3	438	CCGGGCACGTGGTGCTTCAT	TAGCAGCAGATAAACCCAGG
Ep4	424	TTCCGCTCGTGGTGCGAGTGTTC	GAGGTGGTGTCTGCTTGGGTCAG
