# Supplementary figures and images for: Choline Deficiency Causes Colonic Type II Natural Killer T (NKT) Cell Loss and Alleviates Murine Colitis under Type I NKT Cell Deficiency
Source: PLoS One. 2017 Jan 17;12(1):e0169681. doi: 10.1371/journal.pone.0169681 (PMC5241147; doi:10.1371/journal.pone.0169681)

# Fig S1

## (A)

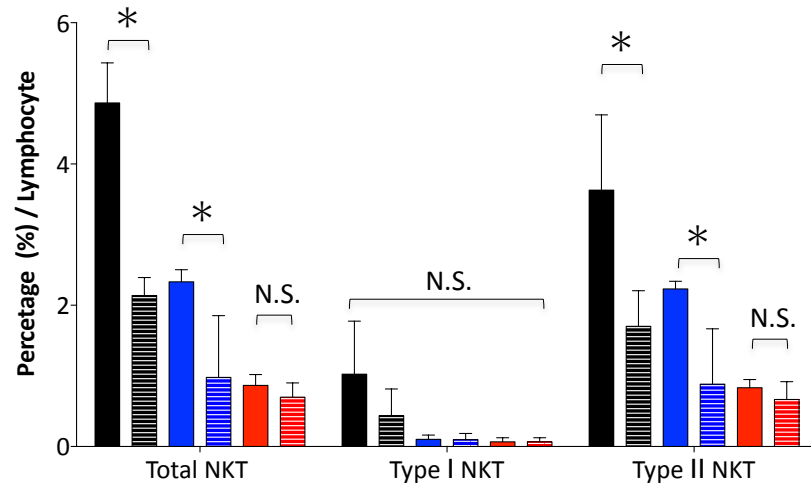

## (B)

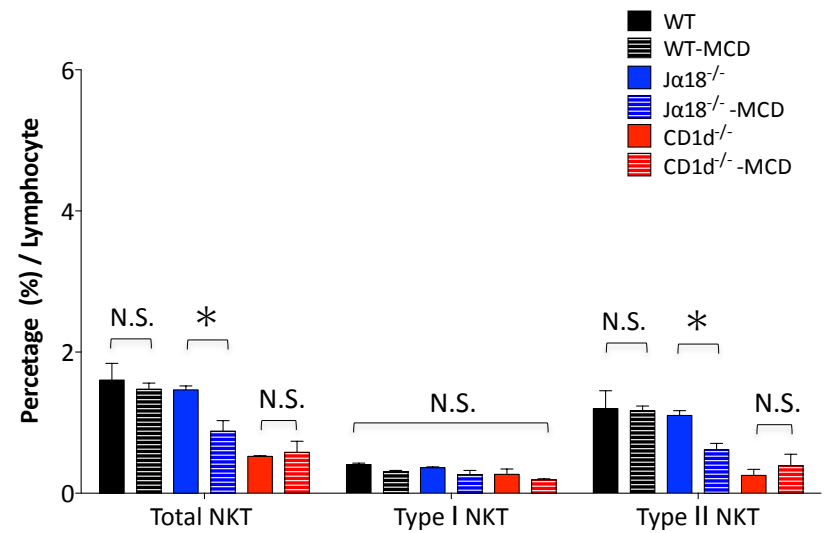

Supplement: S1 Fig — (A) Effects of 1 week of MCD diet on type I and type II NKT cell subsets in the lamina propria of wild-type, Jα18-/-, and CD1d-/- mice before DSS administration. N = 4, *P < 0.05, Student’s t-test. (B) Effects of 1 week of MCD diet on type I and type II NKT cell subsets in the lamina propria of wild-type, Jα18-/-, and CD1d-/- mice after DSS administration. N = 4, *P < 0.05, Student’s t-test. (PDF) [file pone.0169681.s002.pdf]

# Fig S2

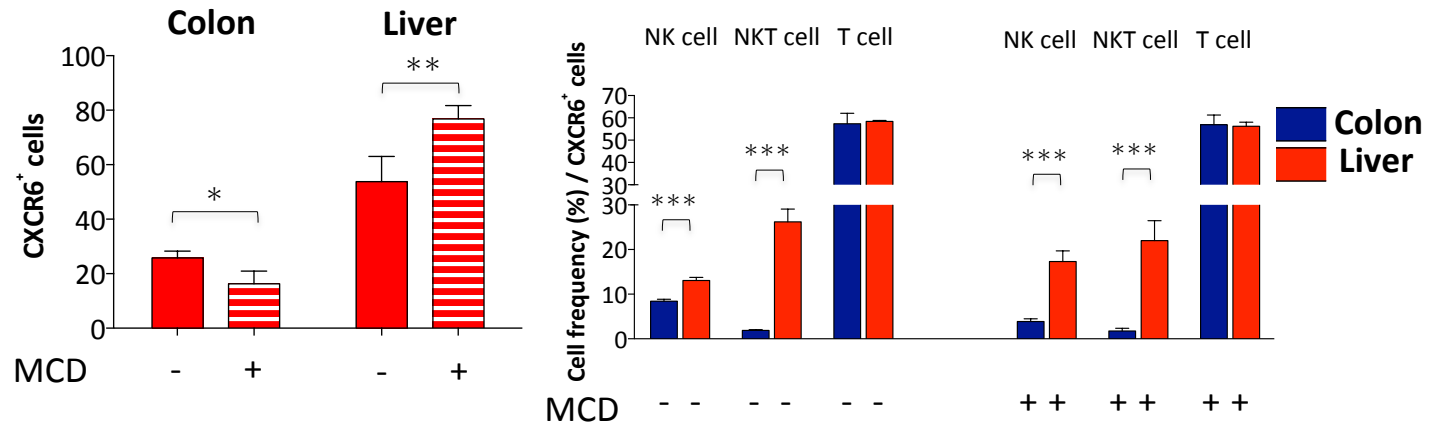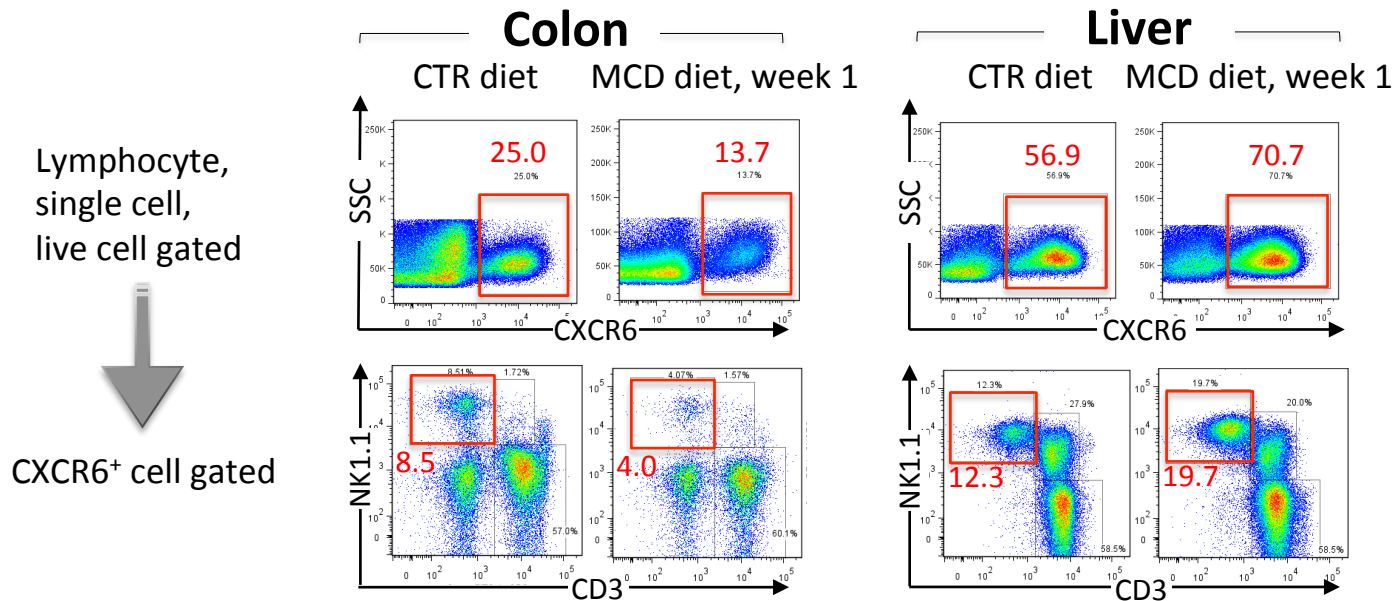

Supplement: S2 Fig — (A-C)Representative flow cytometry plots and frequency of CXCR6+ population in liver and colonic lamina propria in CD1d-/- mice. N = 4, *P < 0.05, **P < 0.01, Student’s t-test. (PDF) [file pone.0169681.s003.pdf]

# Fig S3

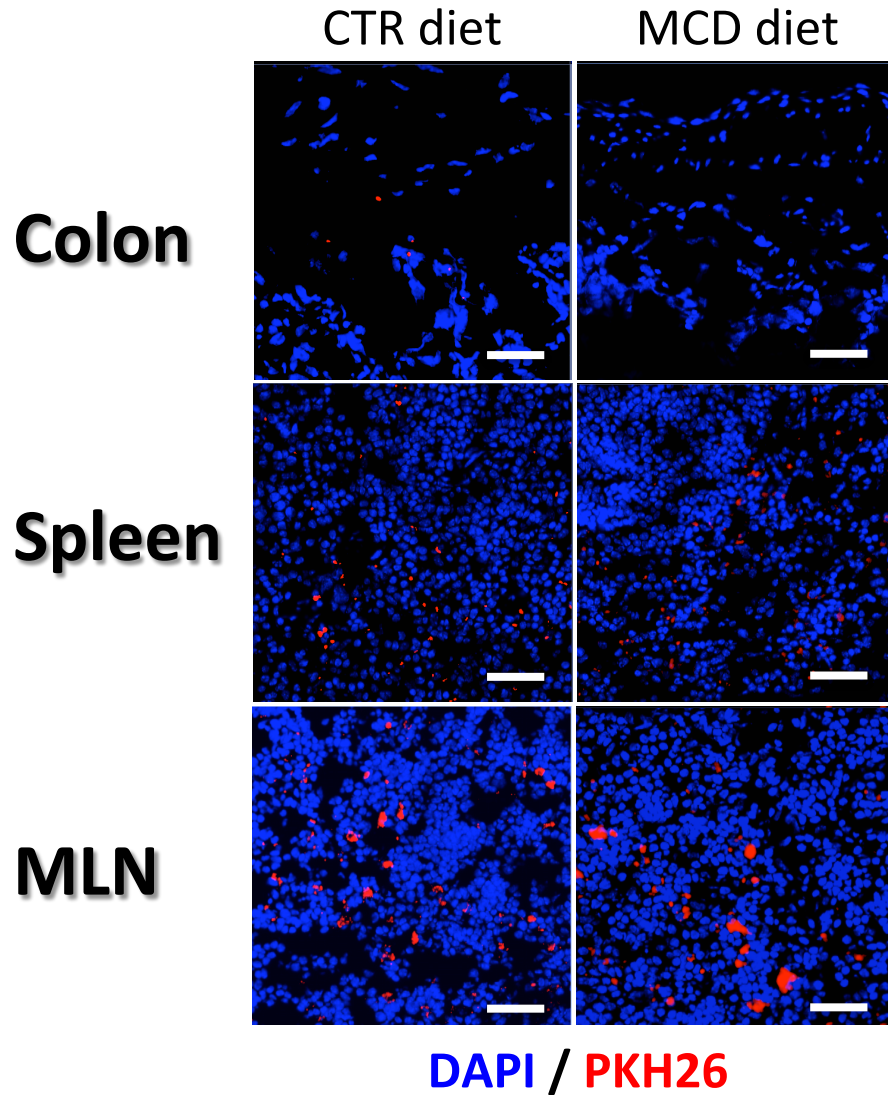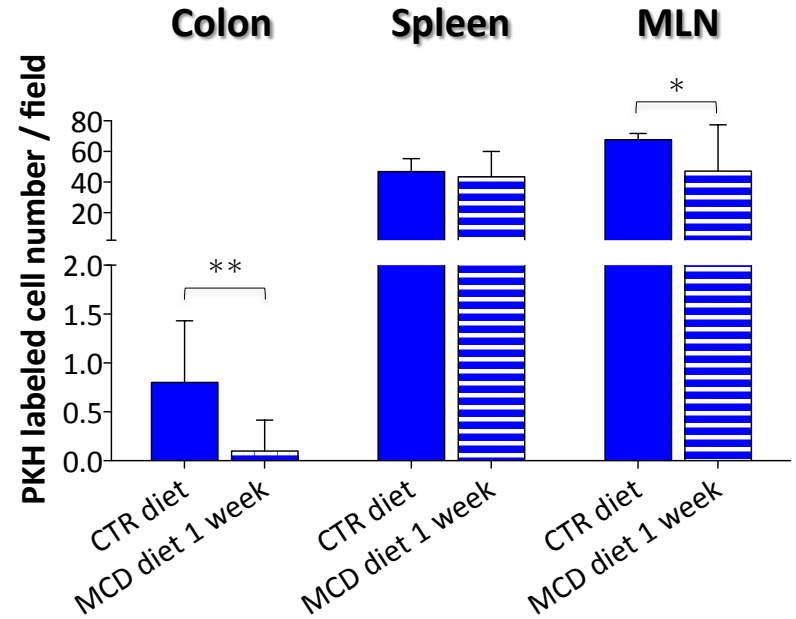

Supplement: S3 Fig — (A-B) Immunofluorescence DAPI staining (×40) of the colon, spleen, mesenteric lymph node from Jα18-/- mice with or without MCD diet at day 7 after injection of PKH-labeled cells. PKH26 (red) and DAPI (blue) were expressed. PKH-labeled cells per field were enumerated. Data from 10 representative fields from four individual mice are plotted as mean ± s.e.m. *P < 0.05, **P < 0.01, Student’s t-test. Scale bar = 50 μm. (PDF) [file pone.0169681.s004.pdf]
