# Supplementary material for: Choline Deficiency Causes Colonic Type II Natural Killer T (NKT) Cell Loss and Alleviates Murine Colitis under Type I NKT Cell Deficiency
Source: PLoS One. 2017 Jan 17;12(1):e0169681. doi: 10.1371/journal.pone.0169681 (PMC5241147; doi:10.1371/journal.pone.0169681)
Supplement: S4 Fig — (PDF) [file pone.0169681.s005.pdf]

# Fig S4

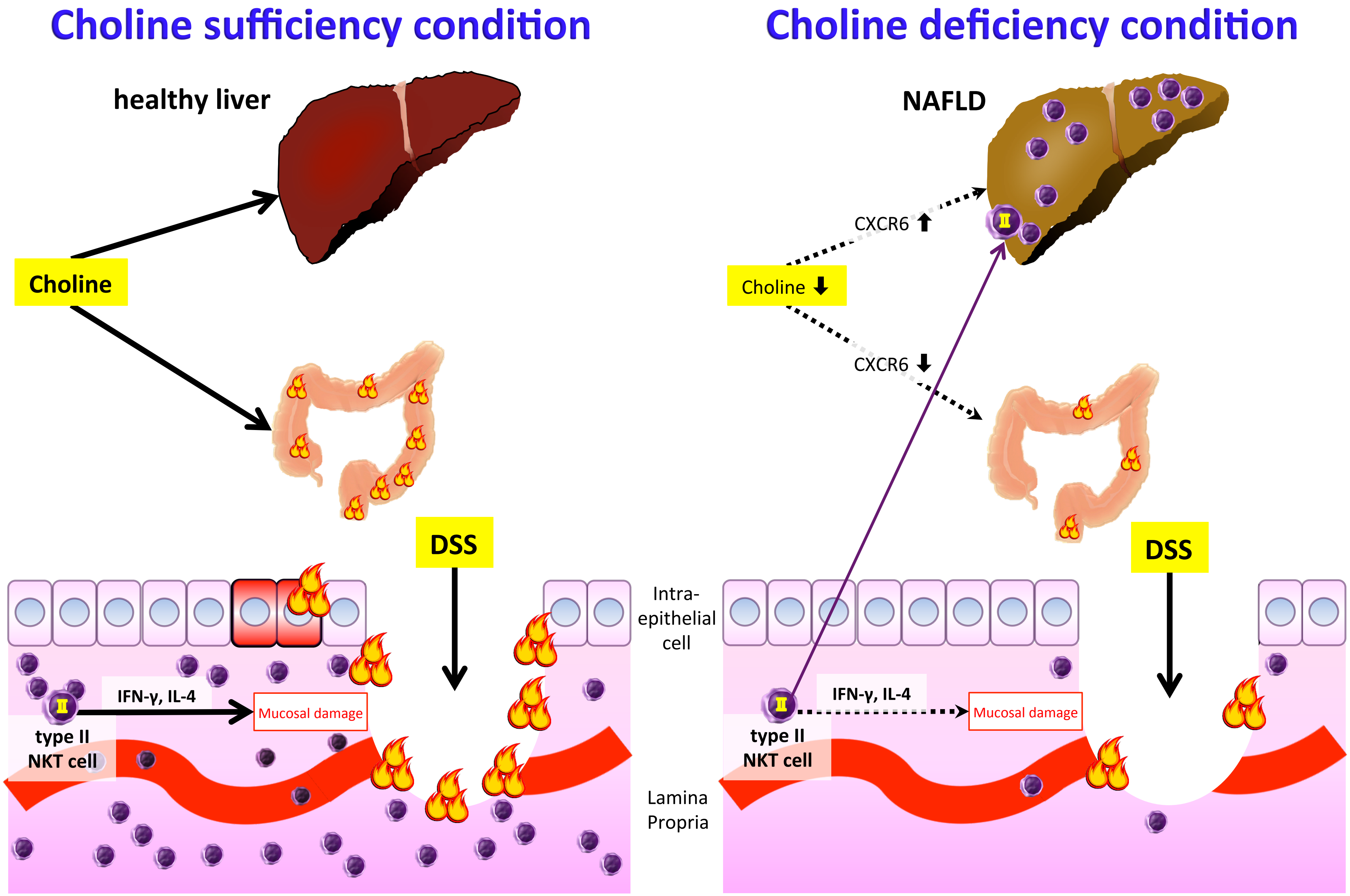
Choline sufficiency condition
Choline deficiency condition
healthy liver
Choline
DSS
Intra-
epithelial
cell
IFN-γ, IL-4
Mucosal damage
type II
NKT cell
Lamina
Propria
NAFLD
CXCR6
Choline
CXCR6
DSS
IFN-γ, IL-4
Mucosal damage
type II
NKT cell
